# Cause rare du syndrome occlusif: la pneumatose kystique intestinale

**DOI:** 10.11604/pamj.2014.19.230.4838

**Published:** 2014-10-30

**Authors:** Hanane Hadj Kacem, Ghiwlane Kharrasse, Tijani El Harroudi

**Affiliations:** 1 Service de Radiologie, Faculté de Médecine et de Pharmacie d'Oujda, 60000, Université Mohamed premier, Oujda, Maroc; 2 Service d'Hépato-Gastroentérologie, Faculté de Médecine et de Pharmacie d'Oujda, Université Mohamed Premier, Maroc; 3 Département de Chirurgie Générale et Cancérologique, Faculté de Médecine et de Pharmacie d'Oujda, Maroc

**Keywords:** Pneumatose kystique, syndrome occlusif, tomodensitométrie (TDM), cystic pneumatosis, occlusive syndrome, computed tomography

## Abstract

La pneumatose kystique intestinale est une Affection rare caractérisée par la présence de kystes gazeux dans la paroi intestinale, pouvant atteindre l'ensemble du tube digestif, avec une prédilection pour l'intestin grêle et le côlon. Le diagnostic est évoqué sur le scanner, permettant d’éviter l'intervention chirurgicale en absence de complications. Les auteurs rapportent une observation rare de pneumatose kystique colique, source de manifestations occlusives imposant l'intervention chirurgicale.

## Introduction

La pneumatose kystique intestinale (PKI) est une entité rare, définie par la présence de kystes de contenu gazeux dans la paroi intestinale [1] qui reste encore mal connue, posant des problèmes diagnostiques et thérapeutiques [1, 2]. Elle peut être primitive ou secondaire associée à de multiples pathologies gastro-intestinales ou autres. Les auteurs rapportent l'observation d'une PKI primitive révélée par un syndrome occlusif aigu.

## Patient et observation

**Figure 1:**
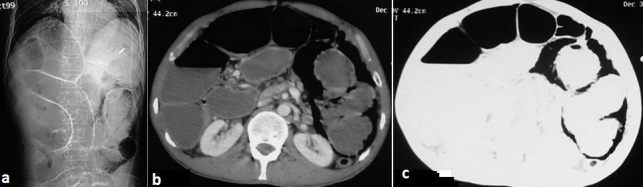
TDM abdominale, Multiples formations gazeuses kystiques au niveau de la paroi grêlique gauche avec distension hydroaérique grêlique en amont. (a): Topogramme; (b): coupes axiales avec injection du produit du contraste; (c): Coupes axiales en fenêtrage pulmonaire

**Figure 2:**
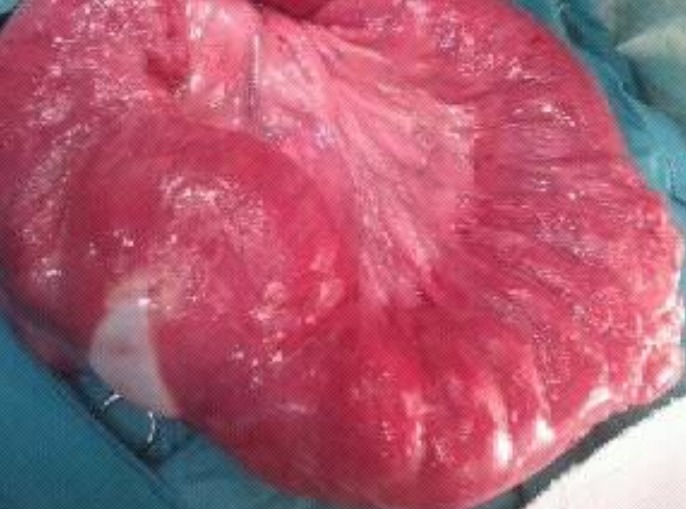
Pièce de résection chirurgicale montrant la pneumatose grêlique

Il s'agit d'un patient âgé de 45ans, admis aux urgences pour douleurs abdominales aigues associées à des vomissements et un arrêt de gaz et du transit depuis trois jours. Le patient n'a pas d'antécédents médicaux ou chirurgicaux particuliers. L'examen clinique retrouve un abdomen augmenté de volume et sensible à la palpation. La radiographie pulmonaire et l'abdomen sans préparation montrent une distension grêlique avec niveaux hydroaériques associée à des images aériques peri-intestinales situées à gauche. Un pneumopéritoine est suspecté et une TDM abdominale est réalisée confirmant la distension grêlique en amont d'une anse légèrement distendue renfermant de multiples formations kystiques pariétales en «grappe de raisin » diffuses, certains kystes sont assez volumineux réduisant la lumière de cette anse et expliquant le syndrome occlusif (Figure 1(a,b,c)). Il n'y avait pas de pneumopéritoine associé. Le bilan biologique retrouve une hyperleucocytose et une hyponatrémie. L'exploration chirurgicale découvere la présence d'une distension grêlique en amont d'une pneumatose kystique au niveau de l'iléon à 10 cm de la valvule de Bauhin (Figure 2). Une résection de 40 cm du grêle avec anastomose termino-terminale est réalisée. Les suites opératoires ont été simples. L'examen anatomo-pathologique de la pièce opératoire a révélé la présence d'une pneumatose kystique sans autre lésion associée.

## Discussion

La pneumatose kystique intestinale (PKI) est une Affection rare caractérisée par la présence de kystes gazeux dans la paroi intestinale, pouvant atteindre l´ensemble du tube digestif, avec une prédilection pour l´intestin grêle et le côlon [1]. Cette affection doit être distinguée nettement de la “pneumatose intestinale aiguë symptôme de pneumatose diffuse ou emphysème intestinal, observée dans les tableaux ischémiques et/ou infectieux sévères lors desquels elle constitue un élément de pronostic très péjoratif, incitant à une chirurgie d´urgence [1, 3]. Sur le plan anatomo-pathologique, Les formations kystiques sont surtout développées dans la sous muqueuse en particulier dans les atteintes coliques, et/ou dans la sous séreuse en particulier au niveau du grêle. L'extension de Ces formations kystiques peut se faire au niveau du mésentère, aux espaces cellulo-graiseux sous péritonéaux du rétropéritoine et des parois abdominales et peut entraimer un pneumopéritoine ou rétro pneumopéritoine bénin [2].

La PKI touche préférentiellement l'homme entre 40 et 50 ans et elle est souvent secondaire ou associée à d'autres pathologies gastro-intestinales (maladie inflammatoire intestinale, ulcère gastroduodénal, sténose pylorique, traumatisme abdominal) ou extra gastro-intestinales (broncho-pneumopathie chronique obstructive, cardiopathies, mucoviscidose, lupus, périartérite noueuse), les formes primitives sont peu fréquents [3]. Chez notre patient, vue l'absence de pathologies associées, nous avons conclu à une pneumatose kystique primitive. Le mécanisme de constitution et d´entretien des kystes gazeux est multifactoriel, faisant intervenir en parties variables plusieurs composantes: perte de l´intégrité de la muqueuse (atteintes infectieuses ou inflammatoires), élévation de la pression endoluminale, modifications de la flore bactérienne, constitutionnelle et/ou acquise et hyperproduction de gaz intestinaux avec perturbations de leurs mécanismes de dégradation [4]. La PKI est généralement pauci-symptomatique. Elle peut être révélée par des signes non spécifiques dans 30% des cas: diarrhée, selles sanglantes ou glaireuses, météorisme, vomissements, constipation, ténesme. L'occlusion intestinale est une complication rare liée au nombre et à la taille volumineuse des kystes pouvant réduire la lumière intestinale et entraînant un syndrome occlusif [5]. D'autres complications liées au volume kystique ont été décrites: volvulus, invagination, perforation, hémorragie [5]. La tomodensitométrie possède une bonne précision diagnostique. Elle révèle des images de densité gazeuse dans la paroi digestive, mieux visibles en fenêtre pulmonaire [6]. Les reconstructions multiplanaires permettent d’étudier précisément la topographie, le volume et l’étendue des kystes. L'association à un pneumopéritoine asymptomatique est quasi pathognomonique [1]. L'examen échographique est non spécifique, il peut suspecter le diagnostic en montrant un amincissement de la paroi intestinale et des échos avec ombre acoustique, réalisant le «signe de l'aurore» [6, 7]. Les examens endoscopiques confirment des kystes sous muqueux, qui produisent un bruit caractéristique lorsqu'ils sont affaissés par la pince à biopsie (popping sound). Il existe un critère diagnostique important permettant de faire le diagnostic différentiel avec la pneumatose intestinale aigué ou les gangrènes intestinales est l'absence d'aéroportie à la tomodensitométrie ou l’échographie [6].

Le traitement reste encore mal codifié, il s'agit le plus souvent d'un traitement médical dont le but est de réduire ou faire disparaître les kystes en en réduisant les bactéries anaérobies qui en sont à l'origine. Il fait appel aux mesures hygiéno-diététiques, une antibiothérapie anti-anaérobie par le métronidazole [7, 3] et l'oxygénothérapie hyperbare. Le traitement chirurgical est indiqué en cas de complications, en cas de symptomatologie rebelle au traitement médical et en cas de pathologie chirurgicale associée, Il consiste à réséquer le segment intestinal atteint par laparotomie ou encore mieux par laparoscopie [7]. Dans notre cas, le patient est opéré vue la sévérité du syndrome occlusif et l'aspect volumineux et sténosant des kystes sur l'imagerie.

## Conclusion

La pneumatose kystique intestinale est une affection peu fréquente, souvent asymptomatique et bénigne. Sa reconnaissance est importante pour éviter d'entreprendre des attitudes thérapeutiques abusives. C'est une cause rare d'occlusion intestinale, dont le diagnostic fait appel essentiellement à la TDM.

## References

[1] Goel A, Tiwari B, Kujur S, Ganguly PK (2011). Pneumatosis cystoides intestinalis. World J Gastroenterol.

[2] Behnoush B, Bazmi B, Mohammadi F, Bazmi E, Dorvashi GA (2009). Pneumatosis Intestinalis: Autopsy Finding. Acta Medica Iranica.

[3] Serraj I, El kihal N, Mohcine R, Essaid A (2006). Pneumatose kystique intestinale avec ascite: association exceptionnelle. Acta Endoscopica..

[4] Rivera Vaquerizo PA, Caramuto Martins A, Lorente García1 MA, Blasco Colmenarejo M, Pérez Flores R (2006). Pneumatosis cystoides intestinalis. Rev Esp Enferm Dig..

[5] Lê P, Benazzouz A, Fritsch L (2003). Une pneumatose kystique colique révélée par un syndrome pseudo occlusif. Ann Chir..

[6] Braham R, Said M, Rehaiem A, Jerbi Omezzine S, Memmi F, Bouabid Z (2004). Imagery of pneumatosis cystica intestinalis. J Chir..

[7] El Bouhaddouti H, Ousadden A, Benjelloun B, Mazaz K, Aït Taleb K (2010). Pneumatose kystique iléale révélée par un volvulus du grêle. Pan Afr Med J..

